# Editorial: Reviews and advances in our understanding of the relationship between the cell cycle, DNA damage and cancer

**DOI:** 10.3389/fcell.2023.1333628

**Published:** 2023-11-16

**Authors:** Damien D’Amours

**Affiliations:** Ottawa Institute of Systems Biology, Department of Cellular and Molecular Medicine, University of Ottawa, Ottawa, ON, Canada

**Keywords:** cell cycle, DNA damage, DNA repair, checkpoints, cancer

The fields of cell cycle and DNA damage have been intertwined since the seminal discovery of checkpoint responses in yeast and human cells exposed to DNA damaging agents. The functional link connecting these processes gained further interest when it became appreciated that patients suffering from cancer predisposition syndromes often carry mutations that impair normal checkpoint activation after exposure to DNA damage. It is now well appreciated that uncontrolled cell cycle progression and loss of genome stability are defining features of cancer cells and the inability to mount an effective cellular response to DNA damage acts as fuel on both of these cancer hallmarks. This Research Topic explores the complex relationship that regulators of cell cycle progression and DNA damage responses have as tumor suppressors and therapeutic targets in oncology. Research teams from around the world bring their unique perspective on this question and have contributed two excellent review articles and three interesting research articles to this Research Topic collection.

Pizzul et al. provide a cogent overview of the complex relationship that connects telomere length regulation to terminal cell cycle arrests, a phenomenon known as replicative senescence. It has been established decades ago that telomeres can be perceived as DNA damage by cells, and preventing this outcome requires specialized DNA replication and end-protection mechanisms. An important question explored in this article is the specific nature of telomeric changes or damages that are responsible for the onset of permanent cell cycle arrest and replicative senescence. Core unresolved issues are discussed, such as the exact threshold length of senescence-inducing telomeres, their overall state in early senescent cells and how homology-directed DNA repair contributes both positively and negatively to creating telomeres that trigger senescence. Importantly, the authors provide a clear overview of the importance of senescence in the development of many pathological conditions in humans, including cancer and aging.

Next, Kim et al. review the multifarious contributions of lamins to cellular homeostasis, aging and cancer. Lamins are among the best-known targets of the cell cycle, being phosphorylated in early mitosis by CDK1, a phenomenon that underpins one of the most dramatic cytological changes experienced by eukaryotic cells: nuclear envelope breakdown. The authors highlight the role of lamins in the maintenance of chromatin position within the nucleus and how this can influence the localization of cellular components–such as telomere proteins and pRb–known to be associated with tumor development in humans. Importantly, they report that mutations in lamins can give rise to nuclear envelope rupture and DNA damage in specific cell types. They also describe how lamin A/C abundance appears to fluctuate widely in different types of cancer cells and suggest that lamins’ contributions to diverse cellular processes should be considered carefully in future oncological research.

In the first research report of this special Research Topic, Sokhi et al. investigate the mechanisms that underpin therapeutic resistance to drugs that target cell cycle and DNA damage checkpoint kinases. In particular, they explore how overexpression of *MYT1*, a key CDK1 inhibitor and G2/M checkpoint effector, can affect the efficacy of drugs that target WEE1 (Adavosertib), WEE1 and MYT1 (PD166285), ATR (AZD6738), and CHK1 (UCN-01) kinases. They show that overexpression of *MYT1* slows down mitotic entry and shortens the duration of mitosis in cancer cells treated with kinase inhibitors. They subsequently explore how these cell cycle effects can lead to a suppression of the cytotoxicity associated with kinase inhibitors. This study provides an excellent example of the complex and sometimes antagonistic relationship that connects cell cycle regulators to DNA damage pathways, and why it is crucial to consider these parameters to prevent therapeutic resistance in the clinic.

In their research article, Yang et al. explore the impact of X-rays on the proliferation of male germinal cells and on the structure of testes in mouse. They specifically focused on the negative effects of irradiation on spermatogonia stem cells–the cell type that generates spermatozoa after a round of meiotic cell division–and how these effects can be mitigated by inhibition of the mTOR signaling pathway. This is an important issue because meiosis is a specialized form of cell division, one that requires the formation of developmentally controlled DNA double-strand breaks, thus making the process sensitive to the formation of additional/unprogrammed lesions in the genome. The authors report that the capacity of testis cells to proliferate effectively after exposure to irradiation is significantly improved by rapamycin-induced mTOR inhibition. Overall, this study provides an interesting first step in the development of novel therapeutic avenues to protect germinal cells from the deleterious effects of radiotherapy treatments used in oncology.

Finally, Sterling et al. report on the intriguing role of BUBR1 in the prevention of DNA damage formation in the brain. BUBR1 is a well-known spindle assembly checkpoint (SAC) protein, and its inactivation is associated with mitotic defects, variegated aneuploidy and microcephaly. Remarkably, the authors noticed that *BubR1* mutant cells accumulated DNA damage in early mitosis, which is an unexpected phenotype for a SAC mutant and suggests the presence of a checkpoint that detects DNA damage specifically in prophase. This study highlights an intriguing crosstalk connecting very different types of checkpoint proteins and how failure to execute the SAC can lead to the activation of the DNA damage checkpoint. This raises interesting questions regarding the interplay of checkpoint mechanisms and how they might compensate for failures in each other to ensure effective cell cycle arrest.

Overall, this Research Topic brings to the forefront the close relationship connecting the cell cycle machinery and the DNA damage response, and how they can often interact to prevent cancer development in humans. Importantly, it is evident from this Research Topic that the relationship connecting the cell cycle and DNA damage machineries can be exploited to treat cancer patients more effectively. The goal of this Research Topic was not to cover the entire breath of this ever-expending area of research, but to provide excellent examples of current questions that motivate contemporary research and new directions for this promising area of research. In this context, we can expect to see many more important studies published on this Research Topic in the coming decade, yielding fascinating studies in both fundamental and clinically relevant areas of cell biology and oncology.

